# Adipose Tissue Lipophilic Index and Risk of Ischemic Stroke—A Danish Case-Cohort Study

**DOI:** 10.3390/nu10111570

**Published:** 2018-10-23

**Authors:** Linda Tram, Stine Krogh Venø, Christina Catherine Dahm, Birthe H. Thomsen, Martin Berg Johansen, Kim Overvad, Erik Berg Schmidt

**Affiliations:** 1 Department of Cardiology, Aalborg University Hospital, Hobrovej 18–22, DK-9100 Aalborg, Denmark; s.venoe@rn.dk (S.K.V.); bht@rn.dk (B.H.T.); ko@ph.au.dk (K.O.); ebs@rn.dk (E.B.S.); 2 Department of Clinical Medicine, Aalborg University, Hobrovej 18–22, DK-9100 Aalborg, Denmark; 3 Department of Public Health, Aarhus University, Bartholins Allé 2, DK-8000 Aarhus C, Denmark; ccd@ph.au.dk; 4 Unit of Clinical Biostatistics, Aalborg University Hospital, Hobrovej 18–22, DK-9100 Aalborg, Denmark; martin.johansen@rn.dk

**Keywords:** lipophilic index, cell membrane fluidity, ischemic stroke, cardiovascular disease, adipose tissue, fatty acids, melting points, case-cohort

## Abstract

Diet may influence the risk of ischemic stroke by several mechanisms. A potential and hitherto unknown mechanism may relate to an effect on the lipophilic index, which is a new and convenient indicator of membrane fluidity. This study investigated the association between the adipose tissue lipophilic index and ischemic stroke and its subtypes. A case-cohort study was conducted based on the Danish cohort study Diet, Cancer, and Health, which includes 57,053 subjects aged 50–64 years at enrolment. A subcohort (*n* = 3500) was randomly drawn from the whole cohort. All ischemic stroke cases were validated and categorized into subtypes. The lipophilic index was calculated based on fatty acid profiles in adipose tissue. Subjects were divided into quintiles and a weighted Cox proportional hazards regression model was used to calculate hazard ratios. After appropriate exclusions, a subcohort of 3194 subjects and 1752 cases of ischemic stroke were included. When comparing the fifth quintile of the lipophilic index with the first quintile, the hazard ratio for ischemic stroke was 0.92 (95% confidence interval 0.75, 1.13) and the trend across quintiles was not statistically significant (*p* = 0.1727). In conclusion, no association was found between the lipophilic index and ischemic stroke or its subtypes.

## 1. Introduction

Ischemic stroke is the second most common cause of death from vascular disease and the third leading cause of disability worldwide [1]. Studies have provided evidence that diet may play an important role in the etiology of ischemic stroke; as such, improving the diet offers an obvious and a modifiable factor for the prevention of ischemic stroke [2]. Consequently, consumption of various types of fatty acids may be of importance for the risk of ischemic stroke. Fatty acid composition in membrane lipids determines membrane fluidity, with the fatty acid availability depending on both fatty acid intake as well as their endogenous synthesis. Previous studies have shown decreased membrane fluidity in patients who have suffered from a stroke [3,4,5]. Decreased membrane fluidity might be a biochemical change related to the development of stroke. Changes in cell membrane fluidity might be modulated through the intake of fatty acids and hence the availability of fatty acids for incorporation into cell membranes [6]. Information on diet can be obtained from interviews and questionnaires, but these results are subject to various forms of bias. In contrast, adipose tissue content of fatty acids is an objective biomarker of long-term dietary intake and endogenous exposure of fatty acids, which may complement dietary data.

The lipophilic index is a weighted mean of the melting points of fatty acids and has been proposed as a simple method of determining overall membrane fluidity. The lipophilic index has previously been found to be associated with myocardial infarction [7], coronary heart disease [8,9], type 2 diabetes mellitus [10], as well as kidney function and blood viscosity [11]. Additionally, other studies of cell membrane fluidity have shown that membrane fluidity was associated with the risk of cardiovascular disease [12,13,14] and therefore could be of relevance for identifying subjects at risk of ischemic stroke.

The aim of the study was to investigate the association between the adipose tissue lipophilic index and incidence of ischemic stroke. Furthermore, we examined whether associations differed between ischemic stroke subtypes.

## 2. Materials and Methods

### 2.1. Study Population

This study was a case-cohort study within the Danish cohort study Diet, Cancer, and Health. Between 1993 and 1997, 160,725 men and women aged 50–64 years were invited to participate in the study and the inclusion criteria were as follows: born in Denmark, living in the Copenhagen or Aarhus areas, and with no previous cancer diagnosis registered in the Danish Cancer Register. A total of 57,053 persons accepted the invitation. A subcohort of 3500 subjects was randomly drawn from within the Diet, Cancer, and Health cohort. 

At baseline, all cohort subjects completed both a lifestyle questionnaire and a food frequency questionnaire [15,16]. Baseline measurements and biological material were obtained by a technician. All subjects provided written informed consent, and the study was approved by the relevant Scientific Ethical Committees and the Danish Data Protection Agency. 

Information on vital status and possible emigration was obtained from the Danish Population Register. The National Patient Register provided information on hospitalization for atrial fibrillation/flutter, which was defined as International Classification of Diseases (ICD) 10 code I48.9 or ICD-8 codes 427.93 or 427.94. From the entire cohort, subjects were excluded due to a prior diagnosis of cancer not registered at baseline (*n* = 569), stroke (*n* = 582), or missing questionnaire (*n* = 42) and, furthermore, cases and subjects from the subcohort were excluded if their fatty acid profile was incomplete (*n* = 335) or there were missing variables (*n* = 44). Previously, knowledge of more than 95% of total fatty acids has been suggested as an adequate proportion to represent a fatty acid composition [6].

### 2.2. Fatty Acid Analysis 

Adipose tissue biopsies were obtained from subcutaneous fat of the upper half of the buttocks of all subjects at baseline. A vacuum tube assembly (Luer lock system, Terumo Corporation, Tokyo, Japan) consisting of a needle (Becton Dickinson Microlance ^TM^3, Becton, Dickinson, and Company, East Rutherford, NJ, USA), a connector (Venoject multi-sample Luer adaptor^®^ Code XX-MN 2000, Terumo Corporation, Tokyo, Japan ), and an evacuated tube (Terumo Venoject VT100 PZ, Terumo Corporation, Tokyo, Japan), in accordance with the method of Beynen and Katan [17], was used. Samples were flushed with nitrogen and stored at −80 °C until final analysis. 

When analyzed, biopsies were thawed, and 1–2 mg of each biopsy was preheated at 50 °C for 10 min. According to International Union of Pure and Applied Chemistry’s Standard Methods for Analysis of Oils, Fats and Derivatives [18], the fat was subsequently dissolved in heptane at 50 °C and 2 g/mol potassium hydroxide in methanol at 50 °C was added for 2 min. to produce fatty acid methyl esters. Fatty acid composition was determined by gas chromatography on a chromatograph (Varian 3900 GC, Middleburg, The Netherlands, with a CP-8400 auto sampler) equipped with a 60 m capillary column with an inner diameter of 0.25 mm and filled with a stabilized cyanopropyl phase, CP-sil 88, and a flame ionization detector, which measures the amount of fatty acid methyl esters with 12 to 24 carbon atoms. Oven temperature was programmed to rise from 90 °C to 220 °C and constant flow was used. Helium was applied as carrier gas. Individual fatty acids were identified using commercially available standards (Nu-chek-Prep, Inc., Elysian, MN, USA), and area percentages of each fatty acid were determined.

The interassay coefficients of variation for the 34 fatty acids determined were below 11.7% for all fatty acids, with an average of 5%.

### 2.3. Calculation of the Lipophilic Index

The lipophilic index was defined as the mean melting points of the fatty acids weighted according to the fatty acid composition (Equation (1)) [7,9].

(1) $$\;\mathrm{LI}\;=\;\frac{\sum_{i\;=\;1}^{k}\mathrm{FA}{\left(\%\right)}_{i}\times {\mathrm{MP}}_{i}}{\sum_{i\;=\;1}^{k}\mathrm{FA}{\left(\%\right)}_{i}}\;$$

where “LI” is the lipophilic index (°C), “FA(%)_i_” is the percentage by weight of the i-th fatty acid (%)], “MP_i_” is the melting point of the i-th fatty acid (°C), “k” is the number of fatty acids included in the calculation, and “i” represents the individual fatty acid [7,9].

Melting points were obtained from LipidBank, the official database of the Japanese Conference on the biochemistry of Lipids [19]. Fatty acids with unknown melting points were omitted and, consequently, assumed to have a melting point equal to the lipophilic index. 

### 2.4. Identification of Outcome

The outcome of the study was ischemic stroke defined according to the World Health Organization’s definition as a rapidly developing disease with focal or global neurologic deficit of vascular origin which lasted more than 24 h [20]. The cohort was linked to the Danish National Patient Register and subjects with ICD-10 discharge codes I60, I61, or I63 or ICD-8 discharge codes 431, 432, 433, 434, 436.01, or 436.90 as either primary or secondary diagnosis were identified as possible stroke cases. All potential stroke cases were reviewed and validated based on clinical appearance, computed tomography scan, magnetic resonance imaging, autopsy records, lumbar punctures, imaging of extra cranial arteries, laboratory tests, electrocardiography, and echocardiography [21]. Ischemic stroke cases were classified according to the Trial of Org 10172 in Acute Stroke Treatment (TOAST) [22] classification: large artery atherosclerosis, cardioembolism, small-artery occlusion, stroke of other etiology, and stroke of undetermined etiology. 

Subjects were followed until the date of a diagnosis of ischemic stroke or censoring, which was the date of death not related to ischemic stroke, date of emigration, or end of follow-up (30 December 2009). 

### 2.5. Statistical Analysis

This case-cohort study assessed the association between the lipophilic index as the exposure and incident ischemic stroke and additionally ischemic stroke subtypes as outcome. 

Since no generally accepted categorization of the lipophilic index existed, the association was explored by means of quintiles. To achieve quintiles representative of the background population, quintiles were defined based on the lipophilic index of the subcohort and cases added accordingly. Weighted Cox proportional hazards analysis was applied to calculate hazard ratios with 95% confidence intervals (CIs), with graphical evaluation of the association performed by exploring cubic splines of the lipophilic index. 

Age was used as the underlying time scale, and to account for the overweight of cases, the Cox regression analyses were weighted using the method of Kalbfleisch and Lawless [23]. Hazard ratios were calculated with the first quintile as a reference. All *p*-values were two-sided and we considered values <0.05 to be statistically significant. All analyses were conducted using Stata 15 (StataCorp LP, College Station, TX, USA).

Adjusted models were constructed to evaluate the association between the lipophilic index and ischemic stroke. Potential confounders were included in three different adjusted models as follows: Model 1a included sex and age at baseline, where sex was included allowing for different baseline hazards while assuming equal associations for exposures [24]. Model 1b was further adjusted for socioeconomic and lifestyle factors including smoking habits (never, former, current <15, 15–25, >25 g tobacco/day), waist circumference adjusted for body mass index (continuous), physical activity (continuous), alcohol intake (continuous), level of education (≤7, 8–10, >10 years) and model 2 additionally included adjustments for medical history of hypercholesterolemia (yes, no, unknown), hypertension (yes, no, unknown), atrial fibrillation/flutter (yes, no), and diabetes mellitus (yes, no, unknown).

A subject was considered to have diabetes mellitus, hypertension, or hypercholesterolemia when the condition was self-reported on the questionnaire or treatment with insulin, antihypertensive, or lipid-lowering drugs was reported. Furthermore, diabetes mellitus was noted if the subject was registered in the National Diabetes Registry at baseline and hypercholesterolemia was noted if the baseline total s-cholesterol measurement was above 5 mmol/L. 

For the restricted cubic splines, continuous variables were modelled using three knots and for comparability purposes, the reference value was the average lipophilic index of the first quintile.

## 3. Results

**Figure 1:**
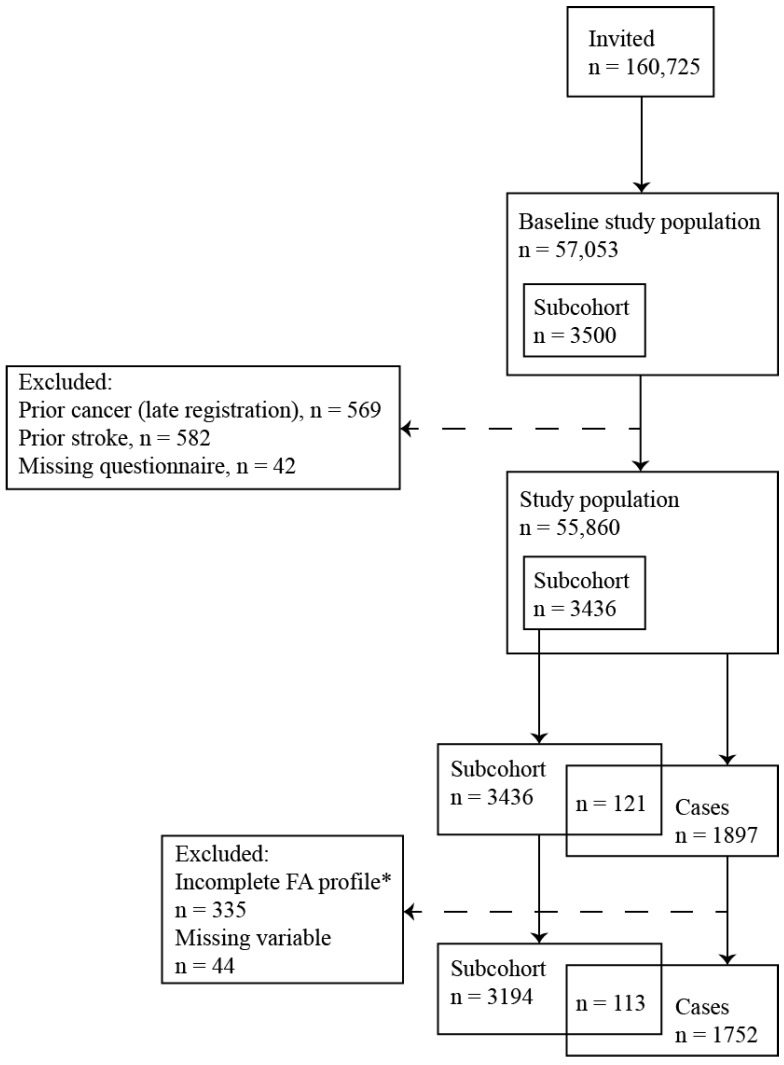
Study flowchart illustrating the cohort size and exclusion criteria applied and hence the case and subcohort size as well as the number of cases within the subcohort. FA = fatty acid. * An incomplete fatty acid profile was defined as a fatty acid content <95% of the total fatty acid composition.

After having applied the exclusion criteria, the study population consisted of 1752 cases of ischemic stroke and a subcohort of 3194 subjects, of whom 113 experienced an ischemic stroke during follow-up. Figure 1 provides a study flowchart. 

Cases were further divided into ischemic stroke subtypes, including 300 cases of large artery atherosclerosis, 779 cases with small-artery occlusion, 99 with cardioembolism, 91 cases of stroke of other etiology, and 483 strokes of unknown etiology (Table 1). Compared to the subcohort, a larger percentage of ischemic stroke cases were older, had a larger waist circumference, were current smokers, had a higher alcohol intake, and had less education and ischemic stroke cases were more likely to have hypertension, diabetes mellitus, hypercholesterolemia, and atrial fibrillation/flutter at baseline. The main differences between the ischemic stroke cases and the subcohort were that more cases had hypertension, were smokers, and had a lower education level.

Furthermore, baseline characteristics when distributed across quintiles of the lipophilic index showed that subjects in the first quintile, who had the lowest lipophilic index, were more likely to have a higher body mass index, a lower level of physical activity, a lower intake of alcohol, hypertension, and atrial fibrillation/flutter than the subjects in the fifth quintile (Table S1: Baseline characteristics of subjects distributed across quintiles). The distribution of ischemic stroke cases and subtypes across quintiles are also displayed in supplementary data (Table S1).

The adipose tissue samples contained up to 34 fatty acids, but melting points were only available for 31 fatty acids, and therefore three fatty acids with unknown melting points were omitted. The sum of fatty acids still included more than 96% of total fatty acids. The included fatty acids were saturated fatty acids 12:0, 14:0, 15:0, 16:0, 17:0, 18:0, 19:0, 20:0, and 22:0, monounsaturated fatty acids 14:1 n-5, 16:1 n-7, 18:1 n-7, 18:1 n-9, 20:1 n-9, 20:1 n-11, 22:1 n-9, and 22:1 n-11, polyunsaturated fatty acids 18:2 n-6, 18:2 n-9, 18:3 n-3, 18:3 n-6, 20:4 n-6, 20:5 n-3, 22:5 n-3, and 22:6 n-3, and trans fatty acids 18:1 ∆6–8, 18:1 ∆-9, 18:1 ∆-10, 18:1 ∆-11, 18:2 ∆ct, and 18:2 ∆tc. The omitted fatty acids were the following polyunsaturated fatty acids 22:4 *n*-6, 20:2 *n*-6, 20:3 *n*-6, and 20:4 *n*-3. A mean melting temperature of trans fatty acids 18:1 ∆6–8 was applied collectively, as differentiation between the trans fatty acids on the gas chromatography was not possible.

**Figure 2:**
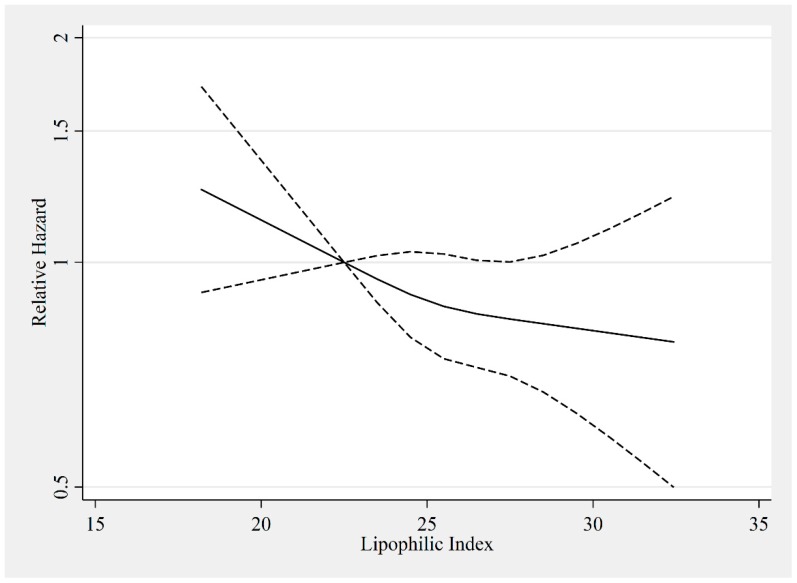
The association between lipophilic index and ischemic stroke illustrated by restricted cubic splines with three knots (solid line) and the 95% confidence interval (dashed line) showing the hazard ratio as a function of the lipophilic index (°C). Model 1b: adjustments for sex, age at baseline, alcohol intake, waistline adjusted for body mass index, education, smoking habits, and physical activity level. The reference value was 22.5 °C.

Figure 2 shows the regression analysis using cubic splines of the lipophilic index as a function of the relative risk of ischemic stroke, which indicated a slight linear statistically insignificant negative tendency. For the analysis, adjustments corresponding to model 1b were used and the reference value was the average lipophilic index of the first quintile, 22.5 °C.

The hazard ratios for an association between the lipophilic index and total ischemic stroke did not indicate a clear trend across quintiles either (Table 2). In the analysis adjusted for potential confounders according to model 1b, the hazard ratio was 0.92 (95% CI: 0.75, 1.13) comparing the fifth quantile with the first quintile. The trend across quintiles was not statistically significant (*p* = 0.1727).

Regarding subtypes of ischemic stroke, there was no clear association between the lipophilic index and large artery atherosclerosis. The lipophilic index was associated with lower rates of small-artery occlusion and cardioembolism, although this association was not statistically significant, and when comparing the fifth quintile with the first quintile the hazard ratios were 0.95 (95% CI: 0.72, 1.24) and 0.69 (95% CI: 0.35, 1.38), respectively.

## 4. Discussion

In this large case-cohort study, which included 1752 cases and a subcohort of 3194 subjects, no clear associations between the adipose tissue lipophilic index and the occurrence of total ischemic stroke as well as subtypes of ischemic strokes could be demonstrated.

When considering the functionality of the cell membrane, it should be able to withstand mechanical stress but, at the same time, not be too rigid to respond to cell stimuli; thus, a u-shaped association between the lipophilic index and ischemic stroke would be reasonable. However, this study showed no association between the adipose tissue lipophilic index and total ischemic stroke, nor its subtypes.

### 4.1. Strengths and Limitations of the Study

Strengths of our study include the study size of 1752 cases of ischemic stroke, with each case having been individually validated independently of the lipophilic index at baseline. Random error is thus not a likely explanation of our study results. Furthermore, each case was categorized according to the TOAST classification, making it possible to investigate stroke according to underlying etiologies. Also, the lipophilic index was calculated based on adipose tissue biopsies, which are objective measures of long-term fatty acid intake and endogenous exposure. Moreover, in the adjusted models, sex, age, and socioeconomic and lifestyle factors, as well as medical history, were added in steps to make it possible to differentiate between the impact of the potential confounders and to ease the interpretation of the results.

The study also had some limitations. In general, the participation in the cohort was only 35% of those invited. However, it is unlikely that questionnaire answers and fatty acid analyses were affected by any systematic error related to the association between lipophilic index and ischemic stroke. Additionally, only middle-aged persons, primarily Caucasians, who had survived without a diagnosis of cancer or ischemic stroke until inclusion were included, hence the results may not be applicable for other age and ethnic groups. The adipose tissue biopsies were only obtained at baseline and changes during follow-up, which would influence the fatty acid composition, were not captured. Furthermore, medical history at baseline did not include pre-hypertensive or pre-diabetic subjects. The untreated subjects may be at higher risk of developing ischemic stroke. However, we did not adjust for changes in medical history in our analysis.

### 4.2. Diet and Ischemic Stroke

Dietary patterns such as Mediterranean, and Dietary Approaches to Stop Hypertension (DASH) have been shown to be associated to the risk of ischemic stroke [2]. A previous study by Tjoenneland et al. [25] has described the diet of the cohort as equivalent to an average Danish diet, which, in 1995, on average contained 114 g/day fats for men and 85 g/day fats for women, with the largest food group being milk and dairy products. Fatty acids from the diet may influence the development of ischemic stroke through several pathways and mechanisms [26]. However, associations between fatty acids and risk of ischemic stroke seem to differ according to the type of fatty acid. Overall, intake of polyunsaturated fatty acids is inversely associated with ischemic stroke risk, while intake of saturated fatty acids is associated with a higher risk of ischemic stroke [27]. The dietary fatty acids are incorporated in cells and tissues after intake but are also stored and released from adipose tissue depending on the availability of the fatty acids. [6]. Hence, the lipophilic index in adipose tissue would be expected to reflect an overall indicator of the cell membrane fluidity. In this study, no association between lipophilic index and ischemic stroke or its subtypes could, however, be demonstrated.

### 4.3. Other Studies of the Lipophilic Index

Only a few other studies regarding the lipophilic index and cardiovascular disease have been published previously. In a case-cohort study of a Dutch population including 2604 subjects and 479 cases with ischemic stroke, no association between the dietary lipophilic index and ischemic stroke or coronary heart disease was found [28], which is in line with our results. The authors explained the neutral results by little variation in the intake of polyunsaturated fatty acids and a low intake of omega-3 fatty acids within the study population, which are known to be associated with lower risk of cardiovascular disease. Furthermore, a high dietary lipophilic index was found to be correlated with higher intakes of saturated and trans fatty acids and lower intake of polyunsaturated fatty acids, suggesting that the lipophilic index reflected the quality of dietary fats [28]. Since our study investigated the same outcome, this may indicate that neither the adipose tissue lipophilic index nor dietary lipophilic index is associated with ischemic stroke.

A Costa Rican matched case-control study by Toledo et al. [7] exploring associations between the adipose tissue lipophilic index and the risk of myocardial infarction included 1672 case-control pairs and analyzed the lipophilic index in adipose tissue and in the diet. The authors found a higher risk of myocardial infarction for the group with the highest adipose tissue lipophilic index compared to the group with the lowest lipophilic index. The lipophilic index in both diet and adipose tissue was associated with higher plasma triglycerides and low-density lipoprotein cholesterol levels. The authors hypothesized that the lipophilic index in adipose tissue and in the diet was associated with myocardial infarction and was possibly mediated through adverse effects on plasma triglycerides and plasma low-density lipoprotein cholesterol. Furthermore, they suggested that the adipose tissue lipophilic index reflected dietary fatty acid composition rather than cell membrane fluidity and that the interpretation of the lipophilic index differed depending on the biological source. A study by Liu et al. [8] analyzed the dietary lipophilic index within a cohort study including 4195 cases with coronary heart disease and the plasma phospholipid lipophilic index within a nested matched case-control study with 1214 pairs. Both indices were associated with a higher risk of coronary heart disease. In a different case-control study, the lipophilic index was determined from 459 cases with coronary heart disease and 879 controls [9]. The lipophilic index was measured in erythrocytes and plasma, but only plasma lipophilic index was associated with a higher risk of coronary heart disease. However, the authors underlined that compared to fatty acids known to be associated with myocardial infarction, the lipophilic index in plasma did not offer additional predictive value of risk.

In the study by Toledo et al. [7], the mean adipose tissue lipophilic index among cases was 23.83 °C and among controls was 23.62 °C, whereas in the present study the mean values were 25.20 °C and 25.22 °C for cases and the subcohort, respectively. The observed difference may reflect different fatty acid intakes in the two study populations, as the study by Toledo et al. [7] was conducted in Costa Rica. 

Since both ischemic heart disease and ischemic stroke are primarily caused by atherosclerosis, the lipophilic index could, based on previous results, a priori be expected to be associated with ischemic stroke. This hypothesis could, however, not be substantiated by findings in this large study of validated cases of total ischemic stroke or subtypes of ischemic stroke, where we found no association between the lipophilic index and incident ischemic stroke. However, our study was an epidemiological study, which can generate hypotheses based on associations but not prove causality. It is still possible that changing a diet from saturated to more unsaturated fatty acids, which would be expected to raise the lipophilic index, as both monounsaturated and polyunsaturated fatty acids have lower melting points than saturated fatty acids [14], could contribute to a reduction in ischemic stroke by such dietary changes. However, the role and potential use of the lipophilic index needs to be documented (or refuted) by future research, which could include different biological material.

## 5. Conclusions

In conclusion, this case-cohort study of 1752 ischemic stroke cases showed no association between the adipose tissue lipophilic index and ischemic stroke or any ischemic stroke subtype. While diet is important for risk of ischemic stroke, this does not seem to be mediated via cell membrane fluidity as measured by the adipose lipophilic index. 

## Figures and Tables

**Table 1:** Baseline characteristics of cases and subcohort. Numerical values are displayed as median and 5th–95th percentiles (P5–P95) and categorical values as percentages and absolute numbers.

Variable [Unit]	Subcohort (*n* = 3194)		Cases (*n* = 1752)	
	Median/%	P5–P95/n	Median/%	P5–P95/n
Lipophilic index [Celsius]	25.23	22.11–28.28	25.23	22.01–28.35
Age at inclusion [years]	56.3	50.7–64.2	58.9	51.0–64.6
Female [% (n)]	46.0	1468	38.1	667
Body mass index [m/kg^2^]	25.8	20.7–33.4	26. 3	21.0–34.9
Waist circumference [cm]	90.0	69.0–111.0	93. 0	72.0–116.0
Physical activity [hours/week]	2.5	0.0–10.5	2.0	0.0–11.0
Smoking status [% (n)]				
- Never	34.7	1107	24.6	431
- Former	29.2	934	26.0	456
- Current <15 g tobacco/day	13.6	434	15.4	269
- Current 15–25 g tobacco/day	15.7	502	23.7	416
- Current >25 g tobacco/day	6.8	217	10.3	180
Alcohol intake [g/day]	13.8	0.8–65.7	14.5	0.5–79.4
Education level [% (n)]				
- ≤7 years	32.9	1050	40.9	717
- 8–10 years	45.0	1437	42.6	746
- >10 years	22.1	707	16.5	289
Hypertension [% (n)]	15.7	502	28.8	505
Diabetes mellitus [% (n)]	2.3	73	4.9	85
Hypercholesterolemia [% (n)]	83.9	2680	86.3	1512
Atrial fibrillation/flutter [% (n)]	0.9	30	1.4	24
Ischemic stroke case [% (n)]	3.5	113		
- Large artery atherosclerosis	16.8	19	17.1	300
- Small-artery occlusion	44.2	50	44.5	779
- Cardioembolism	7.1	8	5.7	99
- Other etiology	3.5	4	5.2	91
- Unknown etiology	28.3	32	27.6	483

**Table 2:** Hazard ratios and 95% confidence intervals for associations for ischemic stroke and subtypes of ischemic stroke. The *p*-values listed are from testing the trend of the hazard ratios. The range of each quintile is given in parentheses (°C).

Model		Q1 (16.4–23.6)	Q2 (23.6–24.7)	Q3 (24.7–25.7)	Q4 (25.7–26.8)	Q5 (26.8–46.4)	*p*-Value
**Ischemic stroke**							
1a ^(1)^	HR	1 (ref.)	0.90	1.05	0.77	0.86	0.0467
	95% CI		0.74, 1.09	0.87, 1.28	0.63, 0.94	0.70, 1.05	
1b ^(2)^	HR	1 (ref.)	0.91	1.06	0.78	0.92	0.1727
	95% CI		0.75, 1.11	0.87, 1.29	0.63, 0.96	0.75, 1.13	
2 ^(3)^	HR	1 (ref.)	0.89	1.07	0.79	0.96	0.4349
	95% CI		0.73, 1.10	0.87, 1.31	0.64, 0.98	0.78, 1.19	
**Large artery atherosclerosis**							
1a ^(1)^	HR	1 (ref.)	0.93	1.51	0.85	1.23	0.4657
	95% CI		0.62, 1.40	1.03, 2.21	0.56, 1.31	0.82, 1.83	
1b ^(2)^	HR	1 (ref.)	0.98	1.56	0.91	1.40	0.1846
	95% CI		0.64, 1.48	1.06, 2.30	0.59, 1.41	0.93, 2.10	
2 ^(3)^	HR	1 (ref.)	0.95	1.55	0.93	1.43	0.1263
	95% CI		0.63, 1.45	1.05, 2.30	0.60, 1.44	0.95, 2.14	
**Small-artery occlusion**							
1a ^(1)^	HR	1 (ref.)	0.90	0.95	0.88	0.91	0.4802
	95% CI		0.70, 1.16	0.74, 1.23	0.68, 1.14	0.70, 1.18	
1b ^(2)^	HR	1 (ref.)	0.90	0.94	0.87	0.95	0.6867
	95% CI		0.69, 1.16	0.72, 1.23	0.67, 1.14	0.72, 1.24	
2 ^(3)^	HR	1 (ref.)	0.88	0.95	0.90	0.99	10.000
	95% CI		0.68, 1.15	0.73, 1.24	0.69, 1.18	0.75, 1.29	
**Cardioembolism**							
1a ^(1)^	HR	1 (ref.)	0.64	0.95	0.74	0.69	0.4065
	95% CI		0.32, 1.31	0.51, 1.77	0.37, 1.45	0.35, 1.35	
1b ^(2)^	HR	1 (ref.)	0.62	0.95	0.71	0.69	0.4357
	95% CI		0.30, 1.29	0.50, 1.81	0.35, 1.44	0.35, 1.38	
2 ^(3)^	HR	1 (ref.)	0.60	0.93	0.69	0.75	0.5822
	95% CI		0.29, 1.26	0.48, 1.81	0.33, 1.42	0.38, 1.49	
**Other etiology**							
1a ^(1)^	HR	1 (ref.)	0.97	0.68	0.43	0.56	0.0107
	95% CI		0.54, 1.76	0.35, 1.30	0.21, 0.88	0.29, 1.08	
1b ^(2)^	HR	1 (ref.)	0.99	0.68	0.44	0.59	0.0201
	95% CI		0.54, 1.79	0.35, 1.31	0.21, 0.92	0.30, 1.16	
2 ^(3)^	HR	1 (ref.)	0.97	0.69	0.44	0.61	0.0269
	95% CI		0.53, 1.76	0.35, 1.34	0.21, 0.92	0.31, 1.20	
**Unknown etiology**							
1a ^(1)^	HR	1 (ref.)	0.91	1.08	0.65	0.72	0.0079
	95% CI		0.67, 1.25	0.80, 1.47	0.47, 0.91	0.52, 1.00	
1b ^(2)^	HR	1 (ref.)	0.93	1.10	0.67	0.78	0.0306
	95% CI		0.67, 1.28	0.80, 1.51	0.47, 0.94	0.55, 1.09	
2 ^(3)^	HR	1 (ref.)	0.91	1.11	0.68	0.83	0.0927
	95% CI		0.65, 1.26	0.80, 1.53	0.48, 0.96	0.59, 1.17	

HR: hazard ratio; 95% CI: 95% confidence interval; Q: quintile; ref.: reference. ^(1)^ Model 1a: Adjusted for sex and age at baseline. ^(2)^ Model 1b: Model 1a + adjustments for alcohol intake, waistline adjusted for body mass index, education, smoking habits, and physical activity level, and ^(3)^ Model 2: Model 1b + adjustments for hypertension, hypercholesterolemia, diabetes mellitus, and atrial fibrillation/flutter.

## Supplementary Materials

The following are available online at http://www.mdpi.com/2072-6643/10/11/1570/s1, Table S1: Baseline characteristics of subjects distributed across quintiles.
