# Supplementary material for: Adipose Tissue Lipophilic Index and Risk of Ischemic Stroke—A Danish Case-Cohort Study
Source: Nutrients. 2018 Oct 23;10(11):1570. doi: 10.3390/nu10111570 (PMC6267621; doi:10.3390/nu10111570)
Supplement: Supplementary file 1 [file nutrients-10-01570-s001.pdf]

nutrients MDPI

| Variable [unit] | Lipophilic index Q1 (n=964) | Q2 (n=955) | Q3 (n=998) | Q4 (n=937) | Q5 (n=979) |
|---|---|---|---|---|---|
| Lipophilic index [Celsius] | 22.80 (20.83 - 23.52) | 24.18 (23.66 - 24.67) | 25.23 (24.77 - 25.67) | 26.22 (25.76 - 26.73) | 27.63 (26.87 - 29.61) |
| Age at inclusion [years] | 57.7 (50.8 - 64.4) | 57.1 (50.7 - 64.4) | 56.8 (50.8 - 64.4) | 57.1 (50.9 - 64.2) | 57.0 (50.7 - 64.5) |
| Female [% (n)] | 71.1 (685) | 52.4 (500) | 41.2 (411) | 32.2 (302) | 20.1 (197) |
| Body mass index [m/kg2] | 26.5 (21.1 - 36.0) | 26.2 (20.8 - 34.2) | 26.0 (21.0 - 33.5) | 25.5 (20.3 - 32.8) | 25.7 (20.6 - 32.0) |
| Waist circumference [cm] | 88.0 (70.0 - 114.0) | 90.0 (69.0 - 114.0) | 91.0 (70.0 - 111.0) | 92.0 (71.0 - 110.0) | 93.0 (71.0 - 110.0) |
| Physical activity [hours/week] | 2.0 (0.0 - 10.0) | 2.0 (0.0 - 11.0) | 2.0 (0.0 - 10.0) | 2.0 (0.0 - 11.5) | 2.5 (0.0 - 12.0) |
| Smoking status [% (n)] | | | | | |
| *- Never* | 34.6 (334) | 31.9 (305) | 30.6 (305) | 28.9 (271) | 29.5 (289) |
| *- Former* | 26.6 (256) | 27.1 (259) | 27.1 (270) | 29.0 (272) | 31.5 (308) |
| *- Current <15 g tobacco/day* | 14.5 (140) | 16.6 (159) | 13.0 (130) | 14.8 (139) | 12.3 (120) |
| *- Current 15-25 g tobacco/day* | 18.7 (180) | 17.1 (163) | 20.0 (200) | 18.5 (173) | 17.8 (174) |
| *- Current >25 g tobacco/day* | 5.6 (54) | 7.2 (69) | 9.3 (93) | 8.8 (82) | 9.0 (88) |
| Alcohol intake [g/day] | 10.1 (0.4 - 65.0) | 13.1 (0.7 - 67.7) | 15.4 (0.7 - 68.6) | 16.4 (1.0 - 70.3) | 18.2 (1.2 - 69.6) |
| Education level [% (n)] | | | | | |
| *- ≤7 years* | 36.3 (350) | 35.6 (340) | 35.4 (353) | 38.7 (363) | 32.6 (319) |
| *- 8-10 years* | 46.5 (448) | 45.9 (438) | 44.5 (444) | 41.0 (384) | 42.8 (419) |
| *- >10 years* | 17.2 (166) | 18.5 (177) | 20.1 (201) | 20.3 (190) | 24.6 (241) |
| Hypertension [% (n)] | 23.4 (226) | 22.0 (210) | 20.6 (206) | 19.4 (182) | 15.5 (152) |
| Diabetes mellitus [% (n)] | 3.0 (29) | 2.6 (25) | 3.5 (35) | 2.8 (26) | 3.9 (38) |
| Hypercholesterolemia [% (n)] | 83.4 (804) | 85.8 (819) | 86.1 (859) | 84.6 (793) | 84.1 (823) |
| Atrial fibrillation/flutter [% (n)] | 1.1 (11) | 1.4 (13) | 1.0 (10) | 1.3 (12) | 0.6 (6) |
| Ischemic stroke case [% (n)] | 36.2 (349) | 35.0 (334) | 39.1 (390) | 33.7 (316) | 37.1 (363) |
| *- Large artery atherosclerosis* | 5.5 (53) | 5.2 (50) | 7.9 (79) | 5.1 (48) | 7.2 (70) |
| *- Small-artery occlusion* | 16.3 (157) | 15.5 (148) | 15.4 (154) | 16.6 (156) | 16.8 (164) |
| *- Cardioembolism* | 2.2 (21) | 1.6 (15) | 2.3 (23) | 2.1 (20) | 2.0 (20) |
| *- Other etiology* | 2.3 (22) | 2.5 (24) | 1.7 (17) | 1.3 (12) | 1.6 (16) |

| | | | | | |
|---|---|---|---|---|---|
| - *Unknown etiology* | 10.0 (96) | 10.2 (97) | 11.7 (117) | 8.5 (80) | 9.5 (93) |

**Table S1** Baseline characteristics of subjects distributed across quintiles. Numerical values are displayed as median and 5th – 95th percentiles and categorical values as percentages and absolute numbers.
